# Function-Driven Design of Lactic Acid Bacteria Co-cultures to Produce New Fermented Food Associating Milk and Lupin

**DOI:** 10.3389/fmicb.2020.584163

**Published:** 2020-11-20

**Authors:** Fanny Canon, Mahendra Mariadassou, Marie-Bernadette Maillard, Hélène Falentin, Sandrine Parayre, Marie-Noëlle Madec, Florence Valence, Gwénaële Henry, Valérie Laroute, Marie-Line Daveran-Mingot, Muriel Cocaign-Bousquet, Anne Thierry, Valérie Gagnaire

**Affiliations:** ^1^INRAE, Institut Agro, STLO, Rennes, France; ^2^INRAE, UR1404 MaIAGE, Jouy-en-Josas, France; ^3^Université de Toulouse, CNRS, INRAE, INSA, TBI, Toulouse, France

**Keywords:** fermented products, lactic acid bacteria, carbohydrates, peptides, amino acids, co-culture, mixed animal-legume resources, closely related phenotypes

## Abstract

Designing bacterial co-cultures adapted to ferment mixes of vegetal and animal resources for food diversification and sustainability is becoming a challenge. Among bacteria used in food fermentation, lactic acid bacteria (LAB) are good candidates, as they are used as starter or adjunct in numerous fermented foods, where they allow preservation, enhanced digestibility, and improved flavor. We developed here a strategy to design LAB co-cultures able to ferment a new food made of bovine milk and lupin flour, consisting in: (i) *in silico* preselection of LAB species for targeted carbohydrate degradation; (ii) *in vitro* screening of 97 strains of the selected species for their ability to ferment carbohydrates and hydrolyze proteins from milk and lupin and clustering strains that displayed similar phenotypes; and (iii) assembling strains randomly sampled from clusters that showed complementary phenotypes. The designed co-cultures successfully expressed the targeted traits i.e., hydrolyzed proteins and degraded raffinose family oligosaccharides of lupin and lactose of milk in a large range of concentrations. They also reduced an off-flavor-generating volatile, hexanal, and produced various desirable flavor compounds. Most of the strains in co-cultures achieved higher cell counts than in monoculture, suggesting positive interactions. This work opens new avenues for the development of innovative fermented food products based on functionally complementary strains in the world-wide context of diet diversification.

## Introduction

*Microorganisms* are ubiquitously encountered as communities on Earth (Widder et al., 2016). They have been selected and widely used in monocultures in various industrial processes such as production of vitamins and organic acids (Piwowarek et al., 2018; De Melo Pereira et al., 2020). A shift is however, observed toward the use of “man-made” bacterial assemblies, here named co-cultures, in which microorganisms can act as a multicellular entity operating using division of labor (Hays et al., 2015; Giri et al., 2019). Division of labor can contribute to speed up transformations, to increase yields in biomass, acidification rates, or production/degradation capacities of molecules of interest. As such, co-cultures are increasingly used to achieve these goals in many areas, as the production of fermented foods and biofuel (Bell et al., 2005; De Melo Pereira et al., 2020). However, designing co-cultures *de novo* to achieve specific functions is still a challenge.

Different design approaches have been used to construct and control some microbial co-cultures with complex functions impossible to get through monoculture-based technologies (Bernstein, 2019). One approach consists in genetically engineering strains to craft metabolic dependencies between them and pooling these strains together in synthetic co-cultures so that the co-culture expresses all the intended functions [see as an example Gilbert et al. (2003)]. Such methods have been applied for the production of molecules of interest such as biofuel (Minty et al., 2013) or pharmaceutics (Ding et al., 2016). Although effective, they, however, implement genetically modified organisms (GMO), and thus cannot be applied for the production of fermented foods, at least in European countries. In traditional fermented foods, fermentation is based on natural complex communities. With the industrialization of food production, starters have been increasingly used, in particular in fermented dairy products, to standardize the final products (Demarigny and Gerber, 2014). However, rational design approaches of “man-made” co-cultures for food purpose have only been scarcely reported. A few systematic iterative approaches have been used to simplify a complex community of a traditional fermented food into a reduced number of strains, which were able to preserve the sensory or other functional properties of the product, e.g., in cheese (Bonaiti et al., 2005; Callon et al., 2011). Co-cultures of bacterial and fungal strains were also recently designed *de novo* by assembling strains from diverse phylogenetic groups, to promote flavor formation and inhibit endogenous undesirable microorganisms in new food products, consisting of emulsions of pea and pea/milk mixes (Ben-Harb et al., 2019). To the best of our knowledge, rational design approaches of co-cultures for food purpose, based on functionally complementary strains have never been reported.

The diversification of food resources is a crucial challenge in the context of sustainable agro-food systems (Ben-Harb et al., 2019). There is actually a growing interest for plant protein-based food products or mixed products that combine animal and plant supplies (Alves and Tavares, 2019; Nicolai, 2019). In the present study, we chose, as an example, a new food that combines bovine milk, with a legume, lupin flour, referred to as milk-lupin mix (MLM). Such a choice was motivated by taking advantages of both resources which contain high nutritional-value proteins and valuable amounts of minerals and vitamins. The main inconvenient of both resources lies in the types of carbohydrates they contain. Milk consumption can actually induce lactose malabsorption and even intolerance (Fassio et al., 2018), while lupin and other legume consumption can generate digestive discomfort due to raffinose-family oligosaccharides (Guillon and Champ, 2002).

Fermentation emerges as a mean to increase the added-value of such new food products, provided that the microbial co-cultures used are properly designed to meet reduction of undesirable carbohydrates (Fritsch et al., 2015; Bartkiene et al., 2016), supply of nutritional requirements, and flavor expectations. Fermentation can also lead to the production of peptides and free amino acids that are involved in texture and/or flavor changes (Lacou et al., 2016) and can promote health benefits via bioactive peptides (Pessione and Cirrincione, 2016). Among the microorganisms used as starter or present as adjuncts in fermented foods, lactic acid bacteria (LAB) play a key role by fermenting carbohydrates into acids, in a wide range of animal and plant-based fermented products, such as yogurt, cheese, salami, sauerkraut, and kimchi (Tamang et al., 2016). The acidification that results from their activity is known to be crucial to limit the growth of pathogen and spoilage microorganisms. Selected LAB strains can hydrolyze the β- and α-galactosides present in milk and legumes, respectively (Teuber, 1995; Gänzle and Follador, 2012), and also possess a complex proteolytic system (Liu et al., 2010). LAB are also important contributors to the typical flavor of fermented foods (Thierry et al., 2015).

The aim of our study was to develop a strategy to design LAB co-cultures capable to both reduce the content in selected carbohydrates and to hydrolyze part of the proteins of the milk-lupin mixes (MLM), to produce peptides and amino acids. Our strategy relied on three successive steps: (i) *in silico* preselection of LAB species that carry genes coding for selected carbohydrate hydrolysis; (ii) *in vitro* screening of strains of the previously selected LAB species for their ability to ferment carbohydrates and hydrolyze proteins, and clustering strains according to their phenotypic similarity, (iii) assembly of functionally complementary strains, randomly sampled from these clusters, to form co-cultures that express the targeted functions.

The approach was assessed by comparing bacterial growth and MLM composition after fermentation either by the designed co-cultures or by the corresponding monocultures. The results validate our strategy, since the designed co-cultures expressed the targeted functions: they decreased the content in the different carbohydrates present in MLM and hydrolyzed proteins. Moreover, co-cultures achieved higher cell counts than monocultures, suggesting positive interactions between strains.

## Materials and Methods

### Design of the LAB Co-cultures

#### *In silico* Screening Based on the Occurrence of Carbohydrate-Related Genes in the Species

The *in silico* screening targeted mesophilic and homofermentative and/or facultatively heterofermentative lactic acid bacteria strains (Vos et al., 2011) on their capability of utilizing the main carbohydrates present in milk (lactose) and legumes (sucrose and raffinose-family oligosaccharides). The complete or draft genome sequences from the Genomes Online Database (GOLD)^1^ (Mukherjee et al., 2017) were used (date of search 11th March 2016). The enzymes were chosen from the KEGG pathway maps for the main lactic acid bacterium *Lactococcus lactis*, to search for genes encoding for proteins homologous to the α-galactosidase of *Lactococcus lactis* KF147 and A12 strains, the α-glycosidase from *L. lactis* NIZO R5 and the β-fructofuranosidase from *Bacillus subtilis* 168. These enzymes are implied in the hydrolysis of carbohydrates from lupin, i.e., raffinose (RAF); stachyose (STA) and sucrose (SUC) (cf Supplementary Figure S1) and the β-galactosidase of *L. lactis* KF147 implied in the hydrolysis of lactose (LAC) from milk. Presence of genes encoding homologous proteins was searched by Tblastn 2.9.0+^2^ with default parameters. Alignments with more than 40% of positive matches on more than 90% of query coverage were used to declare the presence of a targeted gene in a genome.

#### *In vitro* Screening on the Pre-selected Strains

##### Bacterial strains

Ninety-seven mesophilic LAB strains were used to estimate their capability to metabolize carbohydrates and hydrolyze milk and lupin proteins *in vitro* (Supplementary Table S3). 67 lactobacilli strains belonged to nine species from the collection of CIRM-BIA (INRAE Rennes, France) and 30 *Lactococcus lactis* strains from NCDO (Berkshire, United Kingdom), UCMA (Caen, France), and LBAE (Auch, France) collections.

##### Carbohydrate hydrolysis assay

Lactic acid bacteria strains were first reactivated from frozen (−80°C) glycerol stocks in a broth medium, either MRS for lactobacilli or M17-glucose for lactococci at 32°C for 24 h, and cultivated twice on these broths. Lactobacilli strains were then centrifuged at 8,000 *g* × 10 min 20°C and resuspended in API 50 CHL carbohydrate fermentation strips according to the supplier instructions (bioMérieux, Inc., Marcy-l’Etoile, France). A test on stachyose, 6 g/L of API 50 CHL medium, was additionally performed under the same conditions as above. Lactococci strains were inoculated in 96 well plates containing 200 μL of yeast extract medium (pH 6.6) added with 10 g/L of the different carbohydrates and incubated at 30°C for 24 h. Growth was monitored with a Spectramax Plus spectrophotometer (Molecular Devices, Wokingham, United Kingdom) at 580 nm.

##### Protein hydrolysis

Lactic acid bacteria cells were harvested from precultures in the MRS or M17-glucose broth in triplicates for lactobacilli and lactococci respectively, centrifuged at 8,000 *g* × 10 min 20°C and resuspended at 10% (v/v) in a modified API 50 CHL as follows. LAB cells were incubated for 48 h at 30°C. This medium was supplemented with glucose 6 g/L used as the sole carbon source, the yeast extract was diminished to 0.2 g/L to limit the supply in nitrogen compounds that were supplied by either homemade caseinate or lupin isolate (see the paragraph preparation of both isolates below), 5 g/L, or tryptone 5 g/L (BIOKAR Diagnostics, Beauvais, France) this latter being used as a positive control of bacterial growth. The sterile modified API 50 CHL was used as a control for estimating the changes in the nitrogen compounds, peptides and free amino acids released by protein hydrolysis during the incubation (cf biochemical analyses).

#### Clustering of LAB Strains With Similar Phenotypes

The LAB strains were clustered by considering eight phenotypic traits evaluated after *in vitro* analyses: six coded as a binary trait for lactose, galactose, sucrose, fructose, raffinose, and stachyose hydrolysis, and two as continuous traits for the hydrolysis of caseins and lupin isolate (Supplementary Table S3). The data were normalized to give equal weights to all traits before computing Euclidian distances between the phenotypes. The distance matrix was then used to compute a hierarchical classification, using the *hclust* function from the R software^3^ and construct the clusters from that classification. The number of clusters (eight) was chosen to ensure that strains had very similar profiles in each cluster.

### Assembly of LAB Strains Into Co-cultures and the Resulting Fermentation of the MLM

**FIGURE 1:**
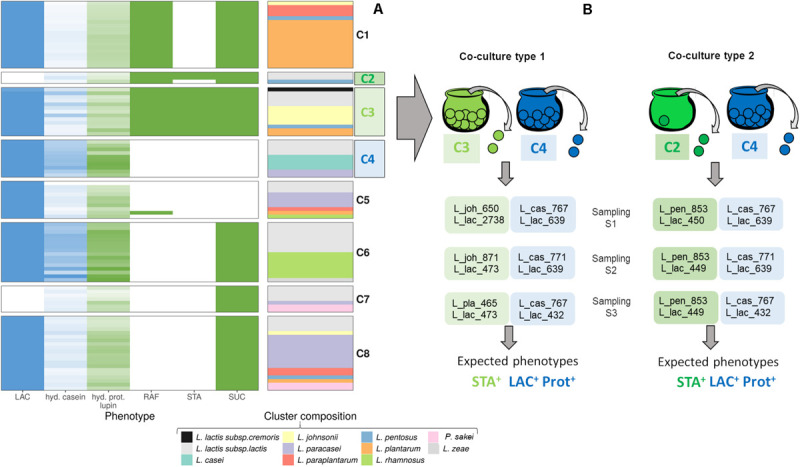
Design of bacterial co-cultures by assembling functionally complementary strains to achieve targeted functions **(A)** Results of the *in vitro* screening and the clustering of LAB strains showing their distribution according to their carbohydrate fermentation and their proteolytic profiles: LAC, lactose; RAF, raffinose; STA, stachyose; SUC, sucrose; hyd.casein and hyd.prot.lupin, proteolytic indices of caseins and lupin proteins. In blue are represented the phenotypes needed to ferment milk-based products, i.e., containing lactose and casein proteins and in green those needed to lupin-based products, i.e., raffinose, stachyose, sucrose and lupin proteins. **(B)** Assembly of the strains from the three selected clusters C2, C3, and C4 to combine strains with complementary phenotypes. Samplings S1 to S3 represent independent variations of co-cultures type 1 Co1 and type 2 Co2 obtained by random sampling of pairs of strains from clusters C2, C3, and C4. Strain names are coded as indicated in Table 2.

Lactic acid bacteria strains were assembled into co-cultures by randomly sampling pairs of strains from selected clusters corresponding to distinct phenotypes (Figure 1). The strains were inoculated at a total count of 10^6^ colony-forming units (CFU)/mL of milk-lupin mixes (MLM) and incubated at 32°C for 24 to 46 h. Non–inoculated MLM was used as a control and incubated under the same conditions.

#### Preparation of Milk-Lupin Mixes (MLM)

The milk was skimmed at the milk platform of INRAE STLO (cream separator Westfalia, Château-Thierry, France) and microfiltered on a pilot fitted with ceramic membranes: (i) to separate microorganisms and milk fat globules (Membralox, 0.8 μm average pore size, Model 7P1940; Pall Exekia, Tarbes, France) at 50°C and (ii) to concentrate the caseins, the main milk proteins (Membralox, 0.1 μm, model 3P1940GL), according to Michalski et al. (2006). Caseins were 2-fold concentrated by diafiltration with osmosed water on the same 0.1 μm membrane, and stored at −20°C.

The milk aqueous phase (permeate), which contains lactose, whey proteins, minerals, small peptides, free amino acids and vitamins, was obtained during the step of microfiltration of milk. It was then ultrafiltered (UF) on an aluminum/zircon membrane (SCT Membralox, 8 kDa average cut-off, type 3P1960) to discard whey proteins. The collected UF permeate was then sterilized by 0.2 μm filtration (Nalgene, Roskilde, Denmark) and stored at 4°C until use.

The lupin flour Protilup 450 (Inveja Lup’ingrédients, France) at 10% (w/v) was suspended into the milk UF permeate to obtain 40 g proteins/L, under stirring for 4 h at room temperature. The suspension was centrifuged 10,000 *g* × 20 min at 20°C to remove insoluble particles. The lupin supernatant and casein micelles were recombined to give a final protein ratio of 50:50. The milk-lupin mix (MLM) was sterilized at 115°C × 20 min and stored at 4°C until use.

#### Preparation of the Caseinate and the Lupin Isolate for Protein Hydrolysis Assays

Both caseinate and lupin isolate were prepared by isoelectric precipitation at pH 4.6 using HCl 1 M, from milk (Le Marchand Farm, Pacé, France) and lupin flour 10% (w/w) (Protilup 450, Inveja Lup’ingrédients, Martigné-Ferchaud, France), respectively. After two washes with pH 4.6 osmosed water, the precipitates were solubilized in pH 6.5 osmosed water, freeze-dried, and stored at 4°C.

#### Bacterial Numeration

Populations were quantified by numeration on agar media on specific media after fermentation: MRS-agar pH 5.4 for lactobacilli and M17-glucose-agar for lactococci (De Man et al., 1960; Terzaghi and Sandine, 1975) and by qPCR using species-specific primers (Supplementary Table S1), which primer efficiency and specificity were checked according to Falentin et al. (2010) (Supplementary Table S2).

#### Biochemical Analyses of Fermented MLM: pH, Carbohydrates, Organic Acids, Global Proteolytic Indices, Free Amino Acids, and Volatile Compounds

##### pH

pH was measured either after 24 h of fermentation (pH meter cyberscan pH110, Eutech instruments, Thermo Fisher Scientific, France) or using a CINAC system (Ysebaert, Frepillon, France) to monitor the acidification profile.

##### Proteolytic indices

The changes in the amount of nitrogen compounds, i.e., peptides and free amino acids present in the MLM fermented or not after 0, 24, and 46 h of incubation were measured in triplicates using the o-phthalaldehyde (OPA) method of Church et al. (1983) adapted to microplate. The proteins were precipitated prior to the assay by half-diluting samples with 2% (w/w) trichloroacetic acid final concentration for allowing the free NH_2_ groups present at the N-terminal extremity of the peptides and amino acids to be preferentially detected by the OPA. The results were expressed as mM equivalent methionine, used as a standard.

##### Free amino acid content

Free amino acid content was determined after deproteinization of the supernatants of MLM fermented or not by 0.23 M sulfosalicylic acid final concentration, incubated for 1 h at 4°C, and centrifuged at 1,000 *g* × 15 min at 20°C to pellet the proteins. The supernatants were filtered through a 0.45-μm pore size membrane (Sartorius, Palaiseau, France), and diluted three times with 0.2 M lithium citrate buffer (pH 2.2) prior to injection. Amino acids were analyzed using cation exchange chromatography on a Biochrom 30 AA analyzer (Biochrom Ltd, Cambridge, United Kingdom) according to Spackman et al. (1958) with lithium citrate buffers as eluents and the ninhydrin as a post-column reaction system.

##### Carbohydrate and organic acid analyses

Carbohydrate and organic acid analyses were performed on the deproteinized samples by sulfosalicylic acid as described above. Lactose, lactate, citrate, and acetate were quantified by high performance liquid chromatography (HPLC) on an Aminex A6 ion-exchange column (Bio-Rad, Hercules, CA, United States) according to Le Boucher et al. (2016). Stachyose, raffinose, verbascose, sucrose, fructose, glucose, galactose, and maltose were quantified by cation exchange chromatography ICS-3000 Dionex (Thermo Electron SA, Courtaboeuf, France) fitted with CarboPac PA1 (4 × 250 mm) analytical column (preceded by a corresponding guard column 50 × 4 mm) according to Aburjaile et al. (2016). Carbohydrate standards (Sigma-Aldrich) were prepared at 2, 5, 10, 20, and 40 mg/L (linearity range).

##### Volatile compounds

Volatile compounds were extracted using a TurboMatrix HS-40 trap automatic headspace sampler and analyzed using a Clarus 680 gas chromatograph coupled to Clarus 600T quadrupole mass spectrometer, operated within a mass range of m/z 29-206 and ionization impact of 70 eV (PerkinElmer, Courtaboeuf, France) as detailed in Pogačić et al. (2015). Volatiles were semi-quantified from the abundance of one specific mass fragment (m/z).

#### Statistical Analyses

Analyses of variance (ANOVA) were performed using the *FactoMineR* package of R software (R Core Team, 2013) to determine whether the strains in monocultures and in co-cultures significantly influenced MLM bacterial cell counts and biochemical composition (residual sugars, organic acids, proteolysis indices, pH values), followed, in case of significant results (*P* < 0.05), by a *post hoc* Fisher’s least significant difference (LSD) test using the *agricolae* R package.

Principal component analysis (PCA) was performed with the biochemical and microbiological data as variables, for all fermented media, using the R package *FactoMineR*.

## Results

### Design of Bacterial Co-cultures, Based on Targeted Functions

Co-cultures of lactic acid bacteria (LAB) were rationally designed by assembling LAB strains, which are functionally complementary to express the targeted functions into the co-cultures, i.e., to ferment carbohydrates and hydrolyze proteins from both milk and lupin. The approach consisted in three successive steps as described below.

#### Step 1: *In silico* Step to Preselect LAB Species With Genes of Interest

The *in silico* analysis was performed on specific functions, i.e., the genes encoding the carbohydrate hydrolases of the available genomes of 19 mesophilic homofermentative LAB species, independently of their taxa and origin. The genes encoding α-galactosidase, α-glucosidase, and β-fructofuranosidase, which hydrolyze lupin carbohydrates (see Supplementary Figure S1), and the gene encoding β-galactosidase, which hydrolyzes lactose, were detected at a low prevalence of 12.4, 13.8, 7.3, and 10.1%, respectively (Table 1). The five species that possessed none of the targeted genes and four others with a very low prevalence of these genes were no longer considered, thus leading to the preselection of ten LAB species.

**TABLE 1:** *In silico* and *in vitro* tests on the ability of mesophilic and homofermentative LAB species to ferment milk lactose and the main three lupin carbohydrates, i.e., raffinose, stachyose and sucrose.

**Lactic acid bacteria species considering the reclassification of lactobacilli by Zheng et al. (2020)**	**Number of strains with specific gene^a^/Number of sequenced strains^b^**				**Number of strains positive for carbohydrate fermentation^c^/number of *in vitro* tested strains**			
	**α-galactosidase gene**	**β-fructofuranosidase gene**	**α-glucosidase gene**	**β-galactosidase gene**	**RAF^+^**	**STA^+^**	**SUC^+^**	**LAC^+^**
***Lacticaseibacillus casei***	9/32	7/32	10/32	3/32	0/4	0/4	0/4	4/4
***Lacticaseibacillus paracasei***	6/51	5/51	4/51	4/51	0/16	0/16	10/16	15/16
***Lacticaseibacillus rhamnosus***	6/40	0/40	5/40	5/40	0/8	0/8	7/8	8/8
***Lacticaseibacillus zeae***	0/2	0/2	1/2	0/2	0/1	0/1	1/1	1/1
***Lactiplantibacillus paraplantarum***	1/2	0/2	1/2	1/2	3/6	0/6	5/6	6/6
***Lactiplantibacillus pentosus***	1/6	1/6	1/6	0/6	3/4	1/4	4/4	3/4
***Lactiplantibacillus plantarum***	11/70	4/70	10/70	7/70	16/17	2/17	16/17	17/17
*Lactobacillus gallinarum*	1/4	0/4	1/4	1/4				
*Lactobacillus gasseri*	3/26	1/26	2/26	3/26				
***Lactobacillus johnsonii***	3/11	1/11	5/11	4/11	6/7	5/7	7/7	7/7
***Lactococcus lactis***	1/75	4/75	7/75	7/75	7/30	7/30	23/30	23/30
*Latilactobacillus curvatus*	0/7	1/7	0/7	0/7				
*Ligilactobacillus ruminis*	1/25	1/25	1/25	0/25				
***Paralactobacillus sakei***	1/4	1/4	1/4	1/4	0/4	0/4	4/4	2/4
Total of *in silico* positive strains among the total number of sequenced strains (%)	44/355 (12.4%)	26/355 (7.3%)	49/355 (13.8%)	36/355 (10.1%)				
**Total for *in vitro* positive strains among the total number of screened strains (%)**					**35/97 (36.1%)**	**15/97 (15.4%)**	**77/97 (79.4%)**	**86/97 (88.7%)**

*^*a*^ The occurrence of strains possessing genes encoding for proteins homologous to four carbohydrate hydrolases are based on homology with the gene encoding for proteins homologous to the α-galactosidase (EC 3.2.1.22) of *Lactococcus lactis* KF147 and A12 strains, the β-galactosidase (EC 3.2.1.23) of *L. lactis* KF147, the α-glucosidase (EC 3.2.1.20/3.2.1.48) from *L. lactis* NIZO R5 and the β-fructofuranosidase (EC3.2.1.26/3.2.1.80) from *Bacillus subtilis* strain 168. ^*b*^ genomes of sequenced strains (complete sequences or drafts) available in the GOLD database the 11th march 2016 (https://gold.jgi.doe.gov/simplesrch); in bold: species selected for *in vitro* screening; The α-galactosidase, β-fructofuranosidase and α-glucosidase are implied in the hydrolysis of carbohydrates from lupin, i.e., raffinose (RAF); stachyose (STA) and sucrose (SUC) (cf Supplementary Figure S1 for more information) and β-galactosidase in the hydrolysis of lactose (LAC) from milk. ^*c*^ The occurrence of strains able to ferment the carbohydrates is based on *in vitro* screening. The names of some lactobacilli species changed. Here are the correspondence between the new and the formerly names, respectively, presented in the table: *Lacticaseibacillus casei – Lactobacillus casei; Lacticaseibacillus paracasei – Lactobacillus paracasei; Lacticaseibacillus rhamnosus – Lactobacillus rhamnosus; Lacticaseibacillus zeae – Lactobacillus zeae; Lactiplantibacillus paraplantarum –Lactobacillus paraplantarum; Lactiplantibacillus pentosus – Lactobacillus pentosus; Lactiplantibacillus plantarum – Lactobacillus plantarum; Lactobacillus gallinarum – Lactobacillus gallinarum; Lactobacillus gasseri – Lactobacillus gasseri; Latilactobacillus curvatus – Lactobacillus curvatus; Ligilactobacillus ruminis – Lactobacillus ruminis; Paralactobacillus sakei – Lactobacillus sakei.* Some lactobacilli species were negative for all the encoding genes, i.e., *Loigolactobacillus coryniformis* formerly named *Lactobacillus coryniformis* (number of sequenced strains = 5), *Companilactobacillus kimchii* formerly named *Lactobacillus kimchii* (*n* = 2), *Liquorilactobacillus mali*, formerly named *Lactobacillus mali* (*n* = 3), *Companilactobacillus paralimentarius* formerly named *Lactobacillus paralimentarius* (*n* = 5) and *Schleiferilactobacillus perolens* formerly named *Lactobacillus perolens* (*n* = 1) and not reported in the table.*

#### Step 2: *In vitro* Step to Evaluate the Ability of the Preselected LAB Species to Degrade Carbohydrates and to Hydrolyze Proteins From Milk and Lupin Resources

A total of 97 strains of the ten remaining LAB species were tested *in vitro* to investigate their ability to degrade carbohydrates and to hydrolyze proteins from both resources (67 *Lactobacillus*-related strains belonging to nine species and 30 *Lactococcus lactis* strains, Supplementary Table S3).

Raffinose was differentially degraded among the tested species (Table 1): either by a very high number of strains per species: 6 out of the 7 *Lactobacillus johnsonii* strains (86%), 3 out of 4 *Lactiplantibacillus pentosus* strains (75%), 16 out of the 17 *Lactiplantibacillus plantarum* strains (94%), or by a lower proportion of strains: 3 out of 6 *Lactiplantibacillus paraplantarum* strains, 7 out of 30 of the *L. lactis* strains (23%), or even by none of the strains tested of *Lacticaseibacillus casei*, *Lacticaseibacillus paracasei*, and *Paralactobacillus sakei.* Stachyose was degraded by only a small fraction of strains of all species, except in *L. johnsonii*, where 5 out of the 7 strains were stachyose-positive (Table 1). Conversely, lactose and sucrose were degraded by most of the tested strains, i.e., 89 and 79%, respectively (Figure 1A and Table 1).

The ability of the strains to hydrolyze or not caseins from milk or proteins from lupin was only evaluated *in vitro* as too many proteolytic enzymes are involved in LAB proteolytic systems (Liu et al., 2010) rendering impossible to preselect LAB species *in silico* on this criterium. The proteolytic activity of the strains was evaluated through the resultant free NH_2_ groups present in the medium after fermentation. For the proteolytic strains, they gave an estimation of the peptides and free amino acids produced in the medium by the proteolytic strains. For the non-proteolytic strains, the initial content of the control medium incubated without bacteria (3.1 ± 0.4 mM eq. Met with caseins and 3.4 ± 0.4 mM eq. Met with lupin proteins) diminished showing a consumption of the peptides and free amino acids already present in the medium. The capability of the strains to hydrolyze proteins varied over a very large range (Figure 1A and Supplementary Table S3), from −3.2 (non-proteolytic strains) to 4.6 (highly proteolytic strains) mM of methionine used as a standard (mM eq. Met) on lupin proteins and −0.8 to 4.1 mM eq. Met on bovine caseins.

#### Step 3: Clustering Step of the LAB Strains With Similar Phenotypes and Assembly of the Functionally Complementary Strains in the Bacterial Co-cultures

Strains were then clustered based on their phenotype, leading to eight clusters, which contained two to seven LAB species (C1 to C8, Figure 1A). Only three clusters, C1, C2, and C3 contained strains capable to degrade raffinose-family oligosaccharides, i.e., raffinose (RAF) and/or stachyose (STA), with a smaller number of strains able to degrade STA, in clusters C2 and C3. One particularity of the clusters was the capability of the strains in 6 clusters out of the 8 to degrade lactose (LAC) or sucrose (SUC). Thus, only the strains clustered in C2 and C7 were not able to degrade LAC, and those in C4 and C5 not SUC. Regarding the proteolytic capacity of the strains, even if it was a continuous feature, some clusters can nevertheless be considered as having high proteolytic activity as in clusters C4 and C6 compared to the other ones.

As the clusters were partially redundant according to the functions targeted, we chose to associate a pair of strains from the cluster C2 (RAF^+^, 2 out of the 3 strains STA^+^ and weakly proteolytic) with a pair of strains from the cluster C4 (only LAC^+^ and highly proteolytic), forming one type of co-culture, named Co2. However, as the cluster C2 contained only three strains, we also associated a pair of strains from the cluster C3 (LAC^+^, RAF^+^ and STA^+^ and moderately proteolytic), with a pair of strains from cluster C4, forming another type of co-cultures, named Co1, which contained some redundancy regarding the proteolytic and lactose hydrolysis functions. To assemble the strains within co-cultures, we randomly sampled one *Lactobacillus-*related strain and one *Lactococcus* strain from each of the selected clusters and combined these pairs of strains to randomly generate three replicates of each type of co-culture, named Co1 (S1, S2, S3) and Co2 (S1, S2, S3) (Figure 1B). In total, 12 different strains were used in at least one of the six co-cultures built (Table 2).

**TABLE 2:** Characteristics of the strains randomly sampled in the three clusters selected after *in silico* and *in vitro* screening and clustering.

**Species**	**Strain number*^a^***	**Code**	**Origin**	**Cluster^b^**	**Carbohydrates*^c^***							**Proteolytic indices*^*d*^* (mM eq Met)**	
					**LAC**	**STA**	**RAF**	**SUC**	**GAL**	**FRU**	**MAL**	**Milk**	**Lupin**
*L. pentosus*	CIRM-BIA853	L_pen_853	Olives	C2	0	0	**1**	**1**	**1**	**1**	**1**	−1.0	−0.7
*L. lactis subsp. lactis*	449 (NCDO 2727)	L_lac_449	Bean	C2	0	**1**	**1**	**1**	0	**1**	0	0.4	−1.5
*L. lactis subsp. lactis*	450 (NCDO 2111)	L_lac_450	Pea	C2	0	**1**	**1**	**1**	**1**	**1**	**1**	−0.2	−1.9
*L. johnsonii*	CIRM-BIA871	L_joh_871	Cantal cheese	C3	**1**	**1**	**1**	**1**	0	**1**	**1**	**1.3**	0.3
*L. johnsonii*	CIRM-BIA650	L_joh_650	Comté cheese	C3	**1**	**1**	**1**	**1**	**1**	**1**	**1**	0.3	0.1
*L. plantarum*	CIRM-BIA465	L_pla_465	Sauerkraut	C3	**1**	**1**	**1**	**1**	**1**	**1**	**1**	−0.4	**2.4**
*L. lactis subsp. lactis*	NCDO 2738	L_lac_2738	Anchu mash	C3	**1**	**1**	**1**	**1**	**1**	**1**	**1**	0.1	−0.6
*L. lactis subsp. lactis*	473 (LBAE-A12)	L_lac_473	Sourdough	C3	**1**	**1**	**1**	**1**	**1**	**1**	**1**	0.3	−1.0
*L. casei*	CIRM-BIA767	L_cas_767	Domiati cheese	C4	**1**	0	0	0	**1**	**1**	0	**2.6**	**4.7**
*L. casei*	CIRM-BIA771	L_cas_771	Domiati cheese	C4	**1**	0	0	0	**1**	**1**	0	**2.4**	**4.4**
*L. lactis subsp. lactis*	432 (UCMA5713)	L_lac_432	Normandy pastures	C4	**1**	0	0	0	**1**	**1**	**1**	**1.5**	**1.4**
*L. lactis subsp. lactis*	CIRM-BIA639	L_lac_639	Tome de Savoie cheese	C4	**1**	0	0	0	**1**	**1**	**1**	**1.7**	**2.7**

*^*a*^ Collections: CIRM-BIA: International Centre for Microbial Resources dedicated to bacteria of food interest, INRAE Rennes, France, http://www6.rennes.inra.fr/cirm_bia_eng/, NCDO, National Collection of Dairy Organisms; now NC of Food Bacteria, Berkshire United Kingdom, UCMA: Université de Caen – Microbiologie alimentaire, Caen, France, and LBAE: Laboratoire de Biotechnologies Agroalimentaire et Environnementale, Auch, France. ^*b*^ Cluster: see Figure 1. ^*c*^ LAC, Lactose; STA, Stachyose; RAF, Raffinose; SUC, Sucrose; FRU, Fructose; GAL, Galactose; MAL, Maltose. Results from API 50 CH gallery (bioMérieux). A value of 1 indicates that the carbohydrate is degraded. ^*d*^ proteolytic index determined by OPA method. Evaluate the capability of the strains to hydrolyze the casein from milk and the lupin protein. Values above 0.5 are in bold.*

### Expression of the Different Functions in the Monocultures

**FIGURE 2:**
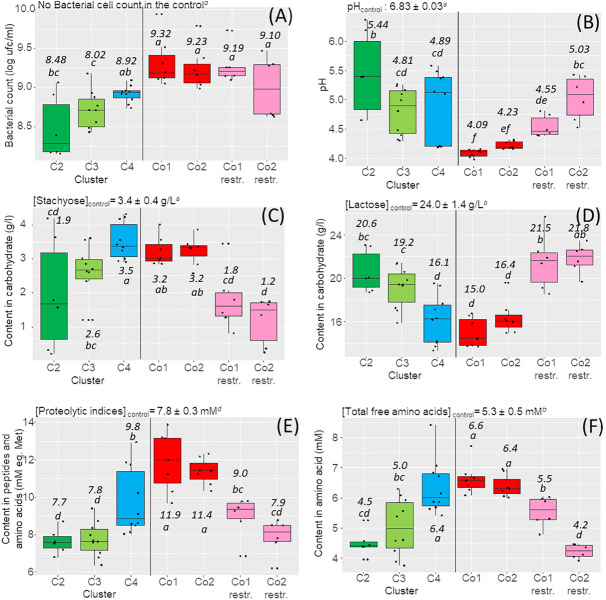
Box plots of the main targeted functions expressed by the strains in the monocultures and in the co-cultures and determined after 24 h fermentation of the milk–lupin mixes (MLM) (*n* = 3 replicates). **(A)** Bacterial cell counts. **(B)** pH. **(C)** Content in stachyose. **(D)** Content in lactose. **(E)** Content in peptides and free amino acids. **(F)** Content in free amino acids. On the left parts of the graphs are shown the results observed in the monocultures: in green, strains of the cluster C2 (LAC^–^/RAF^+^/STA ^+ −^/SUC^+^ and weakly proteolytic), in light green, strains of the cluster C3 (LAC^+^/RAF^+^/STA^+^/SUC^+^and moderately proteolytic), in blue, strains of the cluster C4 (LAC^+^/RAF^–^/STA^–^/SUC^–^ and highly proteolytic). On the right part of the graphs are presented the results observed in the co-cultures Co1 and Co2 in red, the full 4-strain Co1 and Co2, in pink, restricted co-cultures (Co1 *restr*. and Co2 *restr*), i.e., co-cultures depleted in a dominant *L. lactis* strain (CIRM-BIA639 or CIRM-BIA432). The values for the unfermented mix used as the control, are presented on the top of each panel as well as the average values for each cluster and co-cultures on the box plots according to the ANOVA test with significant differences referred with letters.

The 12 strains used in co-culture design were first grown in MLM in monoculture, to check their ability to grow in this medium. They reached cell counts ranging from 8.4 to 9.1 log CFU/mL after 24 h fermentation (Figure 2A green and blue boxes). Concomitantly, the pH of MLM decreased from its initial value of 6.8 to pH 5.0 ± 0.7, with variations depending on the strain (Figure 2B part with in green and blue colors). The functions expressed in MLM by monocultures agreed well with the cluster-associated phenotype. For example, the content in residual stachyose was significantly lower in monocultures of strains from the STA^+^ clusters, compared to strains from the STA^–^ cluster (Figure 2C) with an average decrease from 3.4 g/L in the control to 1.9 g/L in the STA^+^ cluster C2, while no significant difference with the control was observed for the STA^–^ cluster C4. After fermentation, the lactose concentration decreased in cultures of the LAC^+^ clusters C4 and C3, with the respective values of 16.1 and 19.2 g/L compared with those of cluster C2 with 20.6 g/L (Figure 2D). The overall amounts of peptides and free amino acids (proteolytic indices, Figure 2E), and the total amount of free amino acids (Figure 2F), increased only in cultures of strains from the proteolytic cluster C4 comparatively to the initial value of unfermented MLM (control). As expected, the selected strains from clusters C2 and C3 were weakly proteolytic since they did not produce peptides and amino acids, and some strains even decreased their content compared to the control.

### Expression of the Different Phenotypes in the Four-Strain Co-cultures Co1 and Co2

Co-cultures Co1 and Co2 showed globally more homogenous results of carbohydrate and protein degradation compared to monocultures (Figure 2 red color boxes). Moreover, the overall cell counts were significantly higher in co-cultures than in monocultures, regardless of the cluster (9.3 and 9.2 log CFU/mL co-cultures Co1 and Co2 respectively *versus* 8.48 to 8.92 in monocultures). The pH was also lower in MLM fermented by co-cultures with an average pH value of 4.1 in Co1 and 4.2 in Co2 *versus* 4.9 to 5.4 in monocultures (Figure 2B). Proteolytic indices and free amino acid concentrations indicated a higher proteolysis in co-cultures, with an average proteolytic index of 11.6 mM in co-cultures *versus* 7.6 to 9.8 mM in monocultures, and free amino acid concentrations of 6.5 mM *versus* 4.5 to 6.4 mM in co-cultures. However, the main carbohydrate consumed in co-cultures was lactose, as also observed in monocultures of strains from cluster C4. Stachyose (Figure 2C) and raffinose (data not shown) were not degraded although most of the strains from the two STA^+^ clusters C2 and C3 expressed this capability in monocultures.

### Some *L. lactis* Strains Prevent the Co-cultures Co1 and Co2 From Expressing the Targeted Functions

**FIGURE 3:**
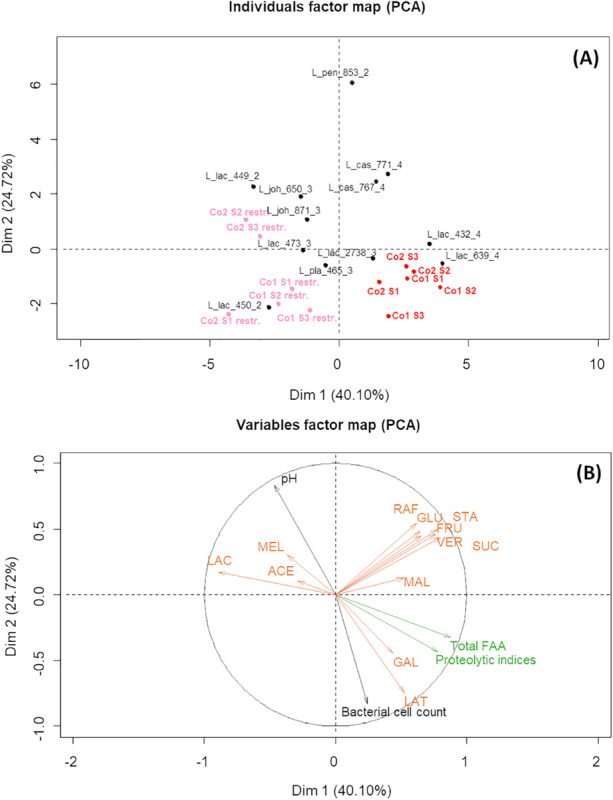
Results of Principal Component Analysis on the biochemical and microbial characteristics of the fermented milk-lupin mixes after 24 h fermentation, showing the first two principal components. PCA individuals factors map **(A)** shows: in red, co-cultures Co1 and Co2, each containing four strains; in pink, restricted co-cultures (Co 1 *restr*. and Co2 *restr*), i.e., co-cultures depleted in a dominant *L. lactis* strains (CIRM-BIA639 or CIRM-BIA432); in black, monocultures of LAB strains. Strain names are coded as indicated in Table 2. Variables factor map **(B)** shows the biochemical and microbial characteristics of the fermented media. Carbohydrates and acids are in orange uppercase: LAC, lactose; SUC, sucrose; RAF, raffinose; STA, stachyose; GAL, galactose; MAL, maltose; GLU, glucose; FRU, fructose; VER, verbascose; MEL, melibiose; LAT, lactic acid; ACE, acetic acid; Total FAA, total free amino acids.

A PCA was built from biochemical and microbial variables describing fermented MLM to compare the global behavior of all monocultures and co-cultures. The factor map showing the first two PCA dimensions shows that Co1 and Co2 (in red) were mainly co-localized with the monocultures of *L. lactis* CIRM-BIA639 and *L. lactis* CIRM-BIA432, the two *Lactococcus* strains of cluster C4 (Figure 3A), suggesting that these strains were dominant in co-cultures. The monocultures of both these lactococci and Co1 and Co2 co-cultures were characterized by a high bacterial cell count, a low pH, a high proteolytic index, and a high lactose consumption (Figure 3B). To determine whether all strains grew in the co-cultures or only these lactococci, we compared the individual cell count in monocultures and in co-cultures (Table 3). The specific cell count of nine out of the 12 strains was most often lower in the co-cultures than in monocultures, suggesting growth inhibition (Table 3). Moreover, the fact that stachyose was not degraded in Co1 and Co2 (Figure 2C) strongly suggests that at least one of the strains from the C2 and C3 clusters were very likely inhibited. Taken together, these results suggest that the two *L. lactis* strains from cluster C4 could be dominant in both co-culture types, where they could have grown and acidified faster than the other strains, thus inhibiting the growth of the latter rendering *de facto* inoperant the metabolic complementarity of the strains within the co-cultures.

**TABLE 3:** Comparison of the LAB cell count in the monoculture vs. the same strains present within the full and restricted co-cultures.

**Co-culture name^a^**	**Cluster^b^**	**Strains present in each co-culture^c^**	**Cell count × 10^9^ (nb of copies/mL or UFC/mL)^d^**			**Cell count ratio**	
			**Monoculture**	**Co-culture**	**Restricted Co-culture**	**Co-culture/monoculture^e^**	**Restricted co-culture/monoculture^e^**
Co1 S1	C3	L_joh_650	0.26	0.02	0.0003	***0.09***	***0.001***
	C3	L_lac_2738	0.56	ND	0.41		0.73
	C4	L_cas_767	0.75	0.95	1.00	1.27	**1.34**
	C3/C4	L_lac_2738 + L_lac_639	1.56	1.75		1.12	
		Sum 4 strains/3 strains	2.57/1.57	2.73	1.41	1.06	0.90
Co1 S2	C3	L_joh_871	0.50	0.30	1.77	***0.60***	**3.55**
	C3	L_lac_473	0.44	ND	0.37		0.84
	C4	L_cas_771	0.72	0.31	1.20	***0.43***	**1.66**
	C3/C4	L_lac_473 + L_lac_639	1.44	0.98		***0.68***	
		Sum 4 strains/3 strains	2.66/1.66	1.58	3.34	***0.59***	**2.01**
Co1 S3	C3	L_pla_465	1.18	0.93	1.98	0.79	**1.68**
	C3	L_lac_473	0.44	ND	0.82		**1.86**
	C4	L_cas_767	0.75	1.35	1.01	**1.81**	**1.35**
	C3/C4	L_lac_473 + L_lac_432	1.49	2.48		**1.67**	
		Sum 4 strains/3 strains	3.41/2.37	4.76	3.81	**1.40**	**1.61**
Co2 S1	C2	L_pen_853	0.33	0.01	0.01	***0.04***	***0.03***
	C2	L_lac_450	1.01	ND	1.31		**1.30**
	C4	L_cas_767	0.75	0.89	1.17	1.19	**1.57**
	C2/C4	L_lac_450 + L_lac_639	2.01	1.14		***0.57***	
		Sum 4 strains/3 strains	3.08/2.08	2.04	2.49	***0.66***	0.81
Co2 S2	C2	L_pen_853	0.33	0.04	0.02	***0.12***	***0.06***
	C2	L_lac_449	0.20	ND	0.28		**1.43**
	C4	L_cas_771	0.72	0.10	0.77	***0.14***	1.07
	C2/C4	L_lac_449 + L_lac_639	1.20	0.78		***0.65***	
		Sum 4 strains/3 strains	2.25/1.25	0.92	1.07	***0.41***	0.86
Co2 S3	C2	L_pen_853	0.33	0.03	0.25	***0.10***	0.75
	C2	L_lac_449	0.20	ND	0.58		**2.98**
	C4	L_cas_767	0.75	0.49	0.58	***0.65***	0.77
	C2/C4	L_lac_449 + L_lac_432	1.24	2.43		**1.96**	
		Sum 4 strains/3 strains	2.31/1.27	2.95	1.41	1.28	1.11

*^*a*^ The names of the co-cultures refers to the Figure 1B. The co-cultures Co1 and Co2 are composed of strains arising from clusters C3 + C4 and C2 + C4, respectively, as shown in Figure 1B. Samplings S1 to S3 represent independent variations of co-cultures obtained by randomly sampling different strains in the corresponding clusters. The term restricted refers to the co-cultures depleted in the dominant lactococci strains L_Lac_639 or L_Lac_432. ^*b*^ Cluster names refer to Figure 1. ^*c*^ Strain names are coded as indicated in Table 2; sum is the sum of cell count of 4 or 3 strains for the full co-cultures or the restricted ones, respectively. ^*d*^ Cell count was evaluated by qPCR quantitation for all lactobacilli species except for *L. casei* for which DNA extraction was not efficient and the cell count was evaluated by numeration. For lactococci, cell count was evaluated by numeration, as lactococci were not distinguishable at the strain level by qPCR with the primers used. In the co-cultures, numeration corresponds to the total lactococci cell counts and in the restricted co-cultures to the only *Lactococcus* strain present. ^*e*^ Ratio are shown in bold is for values above 1.3 and in bold italic for values below 0.7. ND, not determined.*

### Removing the Dominant Strains Allows the Expression of the Targeted Functions in the Co-cultures

To test the hypothesis that *L. lactis* CIRM-BIA639 and CIRM-BIA432 were dominant, we created three-strain co-cultures depleted of the dominant *L. lactis* strain, which we called restricted Co1 and Co2. The restricted co-cultures thus contained only one proteolytic and LAC^+^ strain from cluster C4, either *L. casei* CIRM-BIA767 or *L. casei* CIRM-BIA771. Restricted co-cultures showed a markedly different localization on PCA map compared to the initial co-cultures, due to a different profile of carbohydrate consumption (in pink, and red, respectively, on Figure 3A). Restricted co-cultures contained only between 1.2 and 1.8 g/L of residual stachyose, while only few lactose was consumed with 21.5 and 21.8 g/L of lactose left (Figures 2C,D). Moreover, sucrose was completely consumed in the restricted co-cultures (results not shown) and their final pH was significantly higher (pH 4.5–5.2, Figure 2B).

**FIGURE 4:**
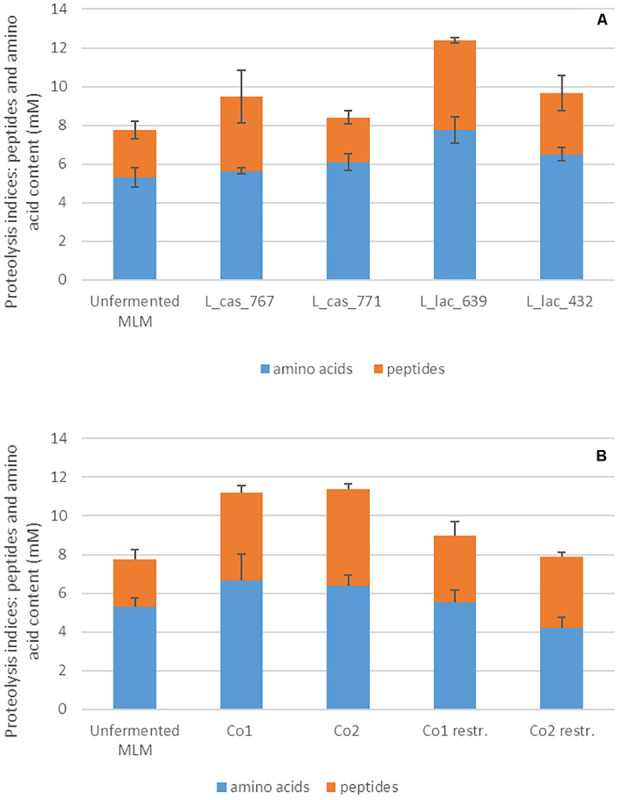
Amounts of peptides and amino acids in the milk-lupin mixes (MLM) fermented or not, **(A)** in the monocultures of the four proteolytic LAB strains sampled from cluster C4, and **(B)** in the co-cultures. The total amount of peptides and amino acids was evaluated by the OPA method and the amino acid amount by amino acid analyzed using cation exchange chromatography. The content in peptides was evaluated by difference between both.

Interestingly, eight out of the ten strains that constituted the restricted co-cultures reached a similar or a higher cell count in the restricted co-cultures than in monocultures (Table 3). Regarding the proteolytic indices and content in amino acids in the restricted co-cultures, they were slightly higher than in control but lower than in the others (Figures 2E,F part in pink color). This can be explained by two factors. First, the two *L. casei* strains from the proteolytic cluster C4 showed a lower proteolysis compared to the two dominant *L. lactis* strains of this cluster (Figure 4A). Second, the non-proteolytic strains, especially related to lactobacilli, reached 1.3 to 3.5-fold higher cell counts in the restricted compared to the 4-strains co-cultures (Table 3). They consumed the amino acids and peptides initially present in MLM and those produced by the proteolytic strains, thus reducing the overall content in peptides and amino acids in MLM fermented by the restricted, compared to the four-strains co-cultures (Figure 4B).

### Co-cultures Also Produced Other Metabolites Such as Volatile Compounds

**FIGURE 5:**
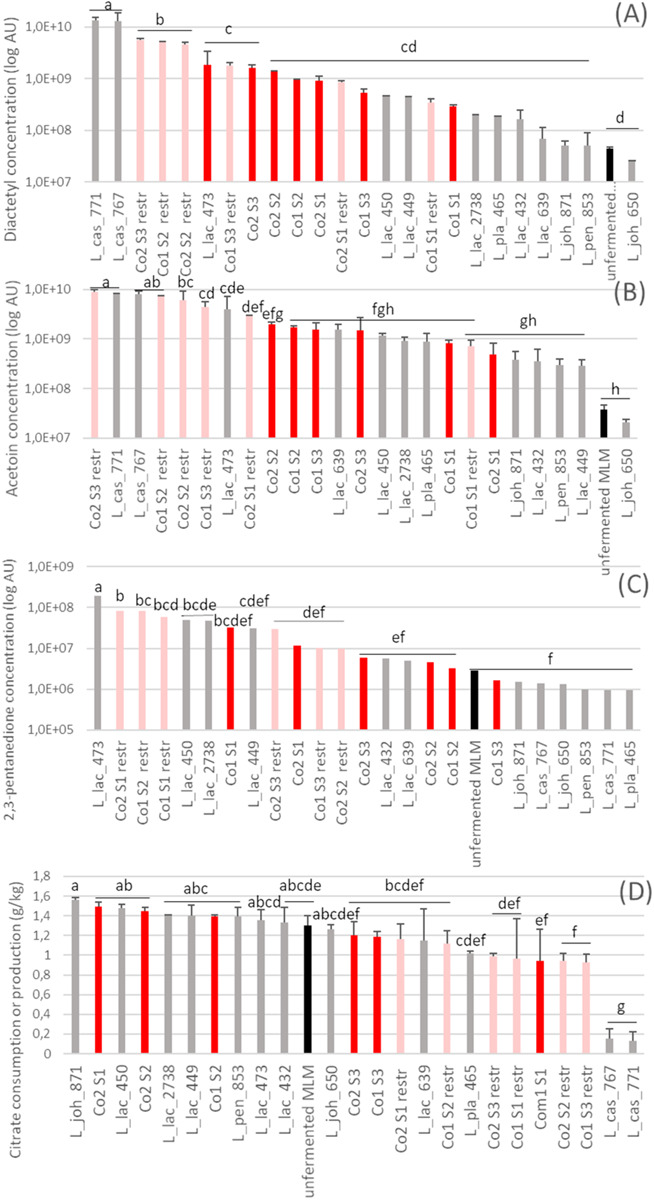
Abundance of three aroma compounds **(A–C)** and concentration of citrate **(D)** after 24 h fermentation of the fermented milk-lupin mixes (MLM). **(A)** 2,3-butanedione (diacetyl), **(B)** 3,2-hydroxybutanone (acetoin) and **(C)** 2,3 pentanedione, in monocultures and co-cultures. In red, 4 strain-co-cultures Co1 and Co2, in pink restricted 3 strain-co-cultures (Co1 *restr*. and Co2 *restr*), i.e., co-cultures depleted in a dominant *L. lactis* strains (CIRM-BIA639 or CIRM-BIA432); co-cultures in gray, monocultures, in black, unfermented MLM (control).

Bacterial metabolism also resulted in the production of other compounds such as volatiles, which were not initially considered for strain selection but gave further information on the behavior of the strains within the co-cultures. Thus, many volatile compounds were identified in fermented MLM. They originated from different pathways and are associated with diverse flavor descriptors (Supplementary Table S4). The content in most of them (22/27) increased during fermentation, whereas hexanal decreased up to 10-fold (Supplementary Table S4). Three volatile compounds associated with desirable flavor in fermented milks are presented as examples in Figure 5. Diacetyl was produced by both *L. casei* strains in a higher amount in monoculture and four out of the six restricted co-cultures than in co-cultures Co1 and Co2 (Figure 5A). A similar pattern was observed for acetoin (Figure 5B). Diacetyl and acetoin derive from citrate conversion (Drinan et al., 1976; Gänzle, 2015), which agreed well the high proportion (90%) of citrate degraded by these two *L. casei* strains in monocultures (Figure 5D). Similarly, 2,3-pentanedione (Figure 5C) was produced by three *L. lactis* strains (473, 450, and 2738) in monocultures and in most of the restricted co-cultures that contained these strains, in concentrations more than 16-fold higher compared to the unfermented MLM. The amount of 2,3-pentanedione, in contrast, did not increase in any of the co-cultures containing these three *L. lactis* strains.

## Discussion

In this study, we developed an original approach to select strains with specific functions and to assemble them into co-cultures designed to ferment new food products that combined milk and lupin. More specifically, we aimed to decrease the concentrations of stachyose and raffinose, which are responsible for digestive discomfort in legume-based products, and that of lactose to alleviate lactose intolerance in dairy products (Guillon and Champ, 2002; Fritsch et al., 2015). We also aimed to hydrolyze proteins for an enhanced production of peptides and free amino acids, which can be involved in the development of flavor and texture, and can modulate health impacts (Thierry et al., 2015; Lacou et al., 2016; Pessione and Cirrincione, 2016). Such a design actually enhanced the conversion of the components from both resources thanks to an assembly of LAB strains to build co-cultures based on their complementary phenotypes, without using GMO, which are not considered as food-grade starters in Europe. The starters used in the food sector also have to minimize organoleptic defaults such as gas production, undesirable texture and flavor, and the development of spoilage and pathogenic microorganisms (Loureiro and Malfeito-Ferreira, 2003; Daly et al., 2010).

The co-cultures were designed using three successive steps which included: a first step to select LAB species *in silico*, a second step to screen *in vitro* a set of strains of the selected species, and the last one to assemble the functionally complementary strains to form co-cultures capable to achieve the targeted functions, i.e., hydrolysis of carbohydrates and proteins of both milk and lupin resources. The *in silico* step allowed reducing the time-consuming step of a classical strain selection based on *in vitro* screening only. In our study, the *in vitro* screening was performed only on the species that possessed genes encoding α- and β-galactosidases or α-glucosidases, the enzymes that hydrolyze lactose and raffinose-family oligosaccharides. Thus, the *in silico* preselection enabled us to reduce the number of LAB species tested *in vitro* from 19 to 10. The results of the *in vitro* screening roughly confirmed the ability of some strains in most *in silico* pre-selected species to ferment the targeted carbohydrates (Table 1). However, the proportion of positive strains *in vitro* did not exactly match that of *in silico* search. For example, a high number of LAB strains were LAC^+^ and SUC^+^ compared to the results expected from the *in silico* search (Table 1). This apparent discrepancy could result from differences in: (i) selection criteria of the set of sequenced strains that were used for the *in silico* search, (ii) variation in the sequence homology between the gene encoding proteins among the species, leading to false-negative results, and (iii) the selection criteria to include strains in the bacterial collections used (Table 1). Actually, the strains present in collections have often been selected for important technological traits related to their main use, i.e., in our case the manufacture of fermented dairy products. *In vitro* screening is mandatory after *in silico* search to investigate the actual ability of LAB strains to degrade the targeted carbohydrates and in particular the raffinose-family oligosaccharides, since enzyme specificity is not accurately predicted from genomic data only and nor the regulation of the enzyme expression. For example, all STA^+^ strains were also able to degrade raffinose but the reverse was not true. As expected, no single strain was able to degrade all the targeted substrates, rendering crucial to assemble functionally complementary strains into co-cultures.

Co-culture design was achieved by assembling strains with complementary phenotypes, a pair of STA^+^/weakly or moderately proteolytic strains and a pair of LAC^+^/highly proteolytic strains (Figure 1), therefore gathering the targeted functions, STA^+^, LAC^+^ and proteolytic, in the designed co-cultures. We ensure some level of functional redundancy by associating pairs of strains from each of the selected clusters. We randomly sampled three pairs of strains from each of the selected clusters to design co-cultures, thus limiting to six the number of experimental co-cultures tested among the 966 possible co-cultures, according to the parameters applied (Figure 1B), to validate the approach of the function-driven design of lactic acid bacteria co-cultures.

The strategy applied gave the expected results, i.e., the targeted functions were reached with both types of co-cultures, rendering effective the functional specialization, as shown in the case of the restricted co-cultures (Figure 3). In the latter, all types of carbohydrates were degraded, demonstrating that strains from the two associated clusters effectively grew. Moreover, some redundancy observed in Co1 co-culture containing strains from cluster C3 (LAC^+^/RAF^+^/STA^+^/SUC^+^ and moderately proteolytic) and cluster C4 (LAC^+^/RAF^–^/STA^–^/SUC^–^ and highly proteolytic) led to a higher release of peptides and amino acids compared with those released in co-cultures Co2 containing strains from clusters C2 (LAC^–^/RAF^+^/STA ^+ −^/SUC^+^ and weakly proteolytic) and C4 (Figure 2 pink boxes). In contrast, for the carbohydrate hydrolysis, we did not observe any change in lactose consumption in both Co1 and Co2 although clusters C3 and C4 were LAC^+^. Several other markers support that different strains effectively contributed to the overall metabolite profiles. For example, volatile metabolites such as diacetyl and 2,3-pentanedione, which were produced only by a few strains in monocultures, were effectively detected at similar amounts in the corresponding co-cultures. In future studies, a higher level of carbohydrate and protein degradation could be obtained by using strains that exhibit the highest degradation capacities.

It is important to highlight that the medium MLM used in the present study was rich enough in nutriments to limit or suppress competition between the strains within co-cultures and thus the expression of the different phenotypes. The cohabitation of strains was nevertheless beyond neutrality, as demonstrated by both positive and negative interactions that occurred in the designed co-cultures. Positive interactions occurred in almost half the restricted co-cultures, in which the cell counts of individual strains in the co-cultures exceeded the cell counts they reached in monocultures (Table 3). This was particularly illustrated in the restricted Co1 S3 (Table 3) since the total count of the three strains present in the co-culture was higher in co-culture than in monoculture, suggesting mutualistic interactions between the strains. These positive interactions could result from cross-feeding and/or sharing of public goods (Canon et al., 2020) likely due to the production of peptides and amino acids by the proteolytic strains and their use by the non-proteolytic ones in complement of those already present in the MLM. This was shown by higher cell counts in co-cultures (mainly the restricted ones), for most of the non-proteolytic strains, than the ones observed in monocultures. In fact, proteolytic strains were shown to contribute to the supply of free amino acids and peptides in yogurt (Bachmann et al., 2015) and in kefir (Dallas et al., 2016), in the form of public goods accessible to interacting strains (Levin, 2014; Giri et al., 2019). Proteolytic strains also reached cell counts similar or higher to the ones observed in monocultures, suggesting that they were not impaired by cheating and may benefit from other cross-feeding mechanisms not elucidated in this study and yet to be investigated.

Negative interactions, such as amensalism, also occurred, notably when a proteolytic and LAC^+^ *L. lactis* strain from the cluster C4 dominated the co-culture and limited in most cases the growth of the other strains present, especially non-*Lactococcus* strains (Figures 2, 3 and Table 3). In these co-cultures, the profile of metabolites, mostly matched the one observed in the monocultures of these dominant *L. lactis* strains (Figure 3). To prevent a possible domination of the co-culture by the fastest fermenting LAB strains, the *in vitro* screening could be refined by considering quantitative data such as the degradation rates, instead of the binary response for carbohydrate degradation retained here.

Globally, our results illustrate how genomic and phenotypic data can be exploited to design co-cultures and produce “new” fermented resources, with different compositions susceptible to modulate the amount of undesirable carbohydrates, their sensory and nutritional properties. With the continuing sharp increase in genome sequencing and improved annotation, it would become possible in the near future to increase the flexibility and modularity of our approach. This will pave the way to select strains based on more complex pathways, for example proteolytic system, citrate utilization, and flavor compound or vitamin production.

## Data Availability Statement

The original contributions presented in the study are included in the article/Supplementary Material, further inquiries can be directed to the corresponding author.

## Author Contributions

All authors listed have made a substantial, direct and intellectual contribution to the work, and approved it for publication.

## Conflict of Interest

The authors declare that the research was conducted in the absence of any commercial or financial relationships that could be construed as a potential conflict of interest.

## Footnotes

1 https://gold.jgi.doe.gov

2 https://blast.ncbi.nlm.nih.gov/Blast.cgi

3 http://www.r-project.org/
