# Supplementary material for: Function-Driven Design of Lactic Acid Bacteria Co-cultures to Produce New Fermented Food Associating Milk and Lupin
Source: Front Microbiol. 2020 Nov 20;11:584163. doi: 10.3389/fmicb.2020.584163 (PMC7717992; doi:10.3389/fmicb.2020.584163)
Supplement: Supplementary file 1 [file Data_Sheet_1.docx]

# Supplementary information (Canon et al.)

**Table S1:** List of primers used for lactic acid bacteria quantification by qPCR

| Target | Primers | Sequences | Targeted genes | Product size (base pair) | References |
| --- | --- | --- | --- | --- | --- |
| *L. johnsonii* | Lj1-F | CACTAGACGCATGTCTAGAG | *16S* | 127 | [1] |
|  | La2-R | AGTCTCTCAACTCGGCTATG | *16S* |  |  |
| *L. paracasei* | TufLcF | GCGATCCAGAACAGGAAAAGG | *tuf* | 130 | [2] |
|  | TufLcR | CGACCAGTGATGGTGAAGACA | *tuf* |  |  |
| *L. paracasei* | Lpar-F | TCCGGGAACTGCTCAGC | *tuf* | 161 | [3] |
|  | Lpar-R | TGTTTCACGAACAGGTG | *tuf* |  |  |
| *L. pentosus* | pentF | CAGTGGCGCGGTTGATATC | *recA* | 218 | [4] |
|  | pREV | TCAGGGTTTCCAAACATCAC | *recA* |  |  |
| *L. plantarum* | planF | CCGTTTATGCGGAACACCTA | *recA* | 318 | [4] |
|  | pREV | TCAGGATTACCAAACATCAC | *recA* |  |  |
| *L. lactis* | Llac-F | TGAAGAATTGATGGAACTCG | *tuf* | 126 | [3] |
|  | Llac-R | CATTGTGGTTCACCGTTC | *tuf* |  |  |

Acccording to Zheng et al [5], here are the corresponding names of the lactobacilli: *L. casei*: *Lacticaseibacillus casei*; *L. paracasei*: *Lacticaseibacillus paracasei*; *L. paraplantarum*: *Lactiplantibacillus paraplantarum*; *L. pentosus*: *Lactiplantibacillus pentosus*; *L. johnsonii*: *Lactobacillus johnsonii*.

**Table S2**: qPCR efficiency and regression coefficients of standard curves

| Target | Primer | Strains | [primers] (nM) | Efficiency | R^2^ |
| --- | --- | --- | --- | --- | --- |
| *L. johnsonii* | Lj1-F/ La2-R | L-joh-650 | F300 R900 | 79.9% | 0.998 |
|  |  | L-joh_871 | F300 R900 | 84.1% | 0.999 |
| *L. paracasei / casei* | TufLcF/ TufLcR | L-cas-771 | F300 R300 | 84.6% | 0.992 |
|  |  | L-cas-767 | F300 R300 | 96.7% | 0.998 |
|  |  | L-par-1517 | F300 R300 | 94.6% | 0.999 |
| *L. paracasei* | Lpar-F/ Lpar-R | L-par-1517 | F300 R300 | 100.2% | 0.997 |
| *L. pentosus* | pentF/ pREV | L-pen-853 | F300 R300 | 86.6% | 0.994 |
| *L. plantarum* | planF/ pREV | L-pla-465 | F300 R300 | 84.5% | 0.987 |
| *L. lactis* | Llac-F/ Llac-R | L-lac-432 | F200 R200 | 101.4% | 0.998 |
|  |  | L-lac-449 | F200 R200 | 98.2% | 0.998 |
|  |  | L-lac-473 | F150 R150 | 119.1% | 0.997 |
|  |  | L-lac-639 | F150 R150 | 109.5% | 0.992 |
|  |  | L-lac-450 | F200 R200 | 98.2% | 0.994 |
|  |  | L-lac-2738 | F200 R200 | 100.2% | 0.968 |

Acccording to Zheng et al [5], here are the corresponding names of the lactobacilli : *L. casei*: *Lacticaseibacillus casei*; *L. paracasei*: *Lacticaseibacillus paracasei*; *L. paraplantarum*: *Lactiplantibacillus paraplantarum*; *L. pentosus*: *Lactiplantibacillus pentosus*; *L. johnsonii*: *Lactobacillus johnsonii*.

**Table S3**: List of the strains tested under *in vitro* conditions and their clustering into groups of similar phenotypes

| Lactic acid bacteria species considering the reclassification of lactobacilli by Zheng et al [5] | | Former names of lactobacilli | | Strain. Number*^a^* | Code | Carbohydrates*^b^* | | | | | | | Proteolytic indices  (mM eq Met)*^c^* | | | Cluster*^d^* |
| --- | --- | --- | --- | --- | --- | --- | --- | --- | --- | --- | --- | --- | --- | --- | --- | --- |
| Genus | Species (subsp) | Genus | Species (subsp) |  |  | LAC | SAC | RAF | STA | FRU | GAL | MAL | | Casein | Lupin |  |
| *Lactiplantibacillus* | *paraplantarum* | *Lactobacillus* | *paraplantarum* | CIRM-BIA670 | L_par_670 | 1 | 1 | 1 | 0 | 1 | 1 | 1 | | -0.4 | -0.4 | C1 |
| *Lactiplantibacillus* | *paraplantarum* | *Lactobacillus* | *paraplantarum* | CIRM-BIA782 | L_par_782 | 1 | 1 | 1 | 0 | 1 | 1 | 1 | | -0.5 | -0.4 | C1 |
| *Lactiplantibacillus* | *paraplantarum* | *Lactobacillus* | *paraplantarum* | CIRM-BIA785 | L_par_785 | 1 | 1 | 1 | 0 | 1 | 1 | 1 | | -0.5 | -0.5 | C1 |
| *Lactiplantibacillus* | *pentosus* | *Lactobacillus* | *pentosus* | CIRM-BIA854 | L_pen_854 | 1 | 1 | 1 | 0 | 1 | 1 | 1 | | -0.3 | -0.3 | C1 |
| *Lactiplantibacillus* | *plantarum* | *Lactobacillus* | *plantarum* | CIRM-BIA466 | L_pla_466 | 1 | 1 | 1 | 0 | 1 | 1 | 1 | | -0.0 | 0.0 | C1 |
| *Lactiplantibacillus* | *plantarum* | *Lactobacillus* | *plantarum* | CIRM-BIA653 | L_pla_653 | 1 | 1 | 1 | 0 | 1 | 1 | 1 | | -0.2 | 0.1 | C1 |
| *Lactiplantibacillus* | *plantarum* | *Lactobacillus* | *plantarum* | CIRM-BIA777 | L_pla_777 | 1 | 1 | 1 | 0 | 1 | 1 | 1 | | -0.3 | -0.1 | C1 |
| *Lactiplantibacillus* | *plantarum* | *Lactobacillus* | *plantarum* | CIRM-BIA845 | L_pla_845 | 1 | 1 | 1 | 0 | 1 | 1 | 1 | | -0.4 | 0.27 | C1 |
| *Lactiplantibacillus* | *plantarum* | *Lactobacillus* | *plantarum* | CIRM-BIA1105 | L_pla_1105 | 1 | 1 | 1 | 0 | 1 | 1 | 1 | | -0.3 | 0.12 | C1 |
| *Lactiplantibacillus* | *plantarum* | *Lactobacillus* | *plantarum* | CIRM-BIA1106 | L_pla_1106 | 1 | 1 | 1 | 0 | 1 | 1 | 1 | | -0.3 | 0.2 | C1 |
| *Lactiplantibacillus* | *plantarum* | *Lactobacillus* | *plantarum* | CIRM-BIA1110 | L_pla_1110 | 1 | 1 | 1 | 0 | 1 | 1 | 1 | | -0.1 | 0.4 | C1 |
| *Lactiplantibacillus* | *plantarum* | *Lactobacillus* | *plantarum* | CIRM-BIA1419 | L_pla_1419 | 1 | 1 | 1 | 0 | 1 | 1 | 1 | | -0.0 | 0.4 | C1 |
| *Lactiplantibacillus* | *plantarum* | *Lactobacillus* | *plantarum* | CIRM-BIA1420 | L_pla_1420 | 1 | 1 | 1 | 0 | 1 | 1 | 1 | | -0.23 | 0.4 | C1 |
| *Lactiplantibacillus* | *plantarum* | *Lactobacillus* | *plantarum* | CIRM-BIA1520 | L_pla_1520 | 1 | 1 | 1 | 0 | 1 | 1 | 1 | | -0.1 | 0.5 | C1 |
| *Lactiplantibacillus* | *plantarum* | *Lactobacillus* | *plantarum* | CIRM-BIA1521 | L_pla_1521 | 1 | 1 | 1 | 0 | 1 | 1 | 1 | | -0.2 | 0.4 | C1 |
| *Lactiplantibacillus* | *plantarum* | *Lactobacillus* | *plantarum* | CIRM-BIA1532 | L_pla_1532 | 1 | 1 | 1 | 0 | 1 | 1 | 1 | | -0.1 | 0.5 | C1 |
| *Lactiplantibacillus* | *plantarum* | *Lactobacillus* | *plantarum* | CIRM-BIA1870 | L_pla_1870 | 1 | 1 | 1 | 0 | 1 | 1 | 1 | | -0.0 | 0.6 | C1 |
| *Lactobacillus* | *johnsonii* | *Lactobacillus* | *johnsonii* | CIRM-BIA868 | L_joh_868 | 1 | 1 | 1 | 0 | 1 | 1 | 1 | | -0.6 | -0.9 | C1 |
| *Lactiplantibacillus* | *pentosus* | *Lactobacillus* | *pentosus* | CIRM-BIA853 | L_pen_853 | 0 | 1 | 1 | 0 | 1 | 1 | 1 | | -0.4 | -0.2 | C2 |
| *Lactococcus* | *lactis (lactis)* |  |  | NCDO2727 | L_lac_449 | 0 | 1 | 1 | 1 | 1 | 0 | 0 | | 0.4 | -1.5 | C2 |
| *Lactococcus* | *lactis (lactis)* |  |  | NCDO2111 | L_lac_450 | 0 | 1 | 1 | 1 | 1 | 1 | 1 | | -0.2 | -1.9 | C2 |
| *Lactiplantibacillus* | *pentosus* | *Lactobacillus* | *pentosus* | CIRM-BIA1490 | L_pen_1490 | 1 | 1 | 1 | 1 | 1 | 1 | 1 | | -0.3 | -0.3 | C3 |
| *Lactiplantibacillus* | *plantarum* | *Lactobacillus* | *plantarum* | CIRM-BIA465 | L_pla_465 | 1 | 1 | 1 | 1 | 1 | 1 | 1 | | -0.3 | 1.9 | C3 |
| *Lactiplantibacillus* | *plantarum* | *Lactobacillus* | *plantarum* | CIRM-BIA1108 | L_pla_1108 | 1 | 1 | 1 | 1 | 1 | 1 | 1 | | -0.2 | 0.3 | C3 |
| *Lactobacillus* | *johnsonii* | *Lactobacillus* | *johnsonii* | CIRM-BIA650 | L_joh_650 | 1 | 1 | 1 | 1 | 1 | 1 | 1 | | 0.4 | -0.1 | C3 |
| *Lactobacillus* | *johnsonii* | *Lactobacillus* | *johnsonii* | CIRM-BIA674 | L_joh_674 | 1 | 1 | 1 | 1 | 1 | 0 | 1 | | -0.1 | -0.2 | C3 |
| *Lactobacillus* | *johnsonii* | *Lactobacillus* | *johnsonii* | CIRM-BIA869 | L_joh_869 | 1 | 1 | 1 | 1 | 1 | 1 | 0 | | 1.8 | 0.9 | C3 |
| *Lactobacillus* | *johnsonii* | *Lactobacillus* | *johnsonii* | CIRM-BIA870 | L_joh_870 | 1 | 1 | 1 | 1 | 1 | 1 | 1 | | -0.2 | -0.7 | C3 |
| *Lactobacillus* | *johnsonii* | *Lactobacillus* | *johnsonii* | CIRM-BIA871 | L_joh_871 | 1 | 1 | 1 | 1 | 1 | 0 | 1 | | 0.5 | -0.3 | C3 |
| *Lactococcus* | *lactis (cremoris)* |  |  | S141 | L_lac_141 | Nd | 1 | 1 | 1 | 1 | 1 | 1 | | 0.5 | -1.0 | C3 |
| *Lactococcus* | *lactis (lactis)* |  |  | A12 | L_lac_473 | 1 | 1 | 1 | 1 | 1 | 1 | 1 | | 0.3 | -1.0 | C3 |
| *Lactococcus* | *lactis (lactis)* |  |  | NCDO2125 | NCDO2125 | 1 | 1 | 1 | 1 | 1 | 1 | 1 | | 1.5 | 01.0 | C3 |
| *Lactococcus* | *lactis (lactis)* |  |  | NCDO2738 | NCDO2738 | 1 | 1 | 1 | 1 | 1 | 1 | 1 | | 0.0 | -0.6 | C3 |
| *Lactococcus* | *lactis (lactis)* |  |  | S645 | L_lac_645 | 1 | 1 | 1 | 1 | 1 | 1 | 1 | | 2.0 | 1.4 | C3 |
| *Lacticaseibacillus* | *casei* | *Lactobacillus* | *casei* | CIRM-BIA667 | L_cas_667 | 1 | 0 | 0 | 0 | 1 | 1 | 0 | | 2.9 | 4.1 | C4 |
| *Lacticaseibacillus* | *casei* | *Lactobacillus* | *casei* | CIRM-BIA767 | L_cas_767 | 1 | 0 | 0 | 0 | 1 | 1 | 0 | | 2.3 | 4.2 | C4 |
| *Lacticaseibacillus* | *casei* | *Lactobacillus* | *casei* | CIRM-BIA769 | L_cas_769 | 1 | 0 | 0 | 0 | 1 | 1 | 0 | | 2.3 | 3.8 | C4 |
| *Lacticaseibacillus* | *casei* | *Lactobacillus* | *casei* | CIRM-BIA771 | L_cas_771 | 1 | 0 | 0 | 0 | 1 | 1 | 0 | | 2.2 | 3.6 | C4 |
| *Lacticaseibacillus* | *paracasei* | *Lactobacillus* | *paracasei* | CIRM-BIA289 | L_par_289 | 1 | 0 | 0 | 0 | 1 | 1 | 1 | | 0.9 | 1.6 | C4 |
| *Lacticaseibacillus* | *paracasei* | *Lactobacillus* | *paracasei (paracasei)* | CIRM-BIA672 | L_par_672 | 1 | 0 | 0 | 0 | 1 | 1 | 1 | | 1.7 | 2.5 | C4 |
| *Lactococcus* | *lactis (lactis)* |  |  | LD61 | L_lac_460 | 1 | 0 | 0 | 0 | 1 | 1 | 1 | | 1.4 | -0.5 | C4 |
| *Lactococcus* | *lactis (lactis)* |  |  | UCMA5713 | L_lac_432 | 1 | 0 | 0 | 0 | 1 | 1 | 1 | | 1.5 | 1.4 | C4 |
| *Lactococcus* | *lactis (lactis)* |  |  | CIRM-BIA236 | L_lac_236 | 1 | 0 | 0 | 0 | 1 | 1 | 1 | | 2.0 | 0.1 | C4 |
| *Lactococcus* | *lactis (lactis)* |  |  | CIRM-BIA639 | L_lac_CIRM639 | 1 | 0 | 0 | 0 | 1 | 1 | 1 | | 1.7 | 2.7 | C4 |
| *Lacticaseibacillus* | *paracasei* | *Lactobacillus* | *paracasei* | CIRM-BIA284 | L_par_284 | 1 | 0 | 0 | 0 | 1 | 1 | 1 | | 0.2 | 1.1 | C5 |
| *Lacticaseibacillus* | *paracasei* | *Lactobacillus* | *paracasei* | CIRM-BIA292 | L_par_292 | 1 | 0 | 0 | 0 | 1 | 1 | 1 | | 0.2 | 1.4 | C5 |
| *Lacticaseibacillus* | *paracasei* | *Lactobacillus* | *paracasei (paracasei)* | CIRM-BIA1869 | L_par_1869 | 1 | 0 | 0 | 0 | 1 | 1 | 1 | | 0.1 | 1.8 | C5 |
| *Lacticaseibacillus* | *paracasei* | *Lactobacillus* | *paracasei (tolerans)* | CIRM-BIA662 | L_par_662 | 1 | 0 | 0 | 0 | 1 | 1 | 0 | | -0.1 | -0.0 | C5 |
| *Lacticaseibacillus* | *rhamnosus* | *Lactobacillus* | *rhamnosus* | CIRM-BIA1436 | L_rha_1436 | 1 | 0 | 0 | 0 | 1 | 1 | 1 | | 0.5 | 1.0 | C5 |
| *Lactiplantibacillus* | *paraplantarum* | *Lactobacillus* | *paraplantarum* | CIRM-BIA775 | L_par_775 | 1 | 0 | 0 | 0 | 1 | 1 | 1 | | -0.5 | -0.4 | C5 |
| *Lactiplantibacillus* | *plantarum* | *Lactobacillus* | *plantarum* | CIRM-BIA1524 | L_pla_1524 | 1 | 0 | 1 | 0 | 1 | 1 | 1 | | -0.2 | 0.5 | C5 |
| *Lactococcus* | *lactis (lactis)* |  |  | S175 | L_lac_482 | 1 | 0 | 0 | 0 | 1 | 1 | 1 | | -0.8 | -3.2 | C5 |
| *Lactococcus* | *lactis (lactis)* |  |  | CIRM-BIA44 | L_lac_CIRM44 | 1 | 0 | 0 | 0 | 1 | 1 | 1 | | 0.2 | -0.6 | C5 |
| *Lactococcus* | *lactis (lactis)* |  |  | CIRM-BIA86 | L_lac_CIRM86 | 1 | 0 | 0 | 0 | 1 | 1 | 0 | | 0.3 | -0.7 | C5 |
| *Lacticaseibacillus* | *rhamnosus* | *Lactobacillus* | *rhamnosus* | CIRM-BIA 774 | L_rha_774 | 1 | 1 | 0 | 0 | 1 | 1 | 1 | | 3.2 | 4.5 | C6 |
| *Lacticaseibacillus* | *rhamnosus* | *Lactobacillus* | *rhamnosus* | CIRM-BIA 776 | L_rha_776 | 1 | 1 | 0 | 0 | 1 | 1 | 1 | | 1.6 | 2.2 | C6 |
| *Lacticaseibacillus* | *rhamnosus* | *Lactobacillus* | *rhamnosus* | CIRM-BIA780 | L_rha_780 | 1 | 1 | 0 | 0 | 1 | 1 | 1 | | 2.2 | 2.7 | C6 |
| *Lacticaseibacillus* | *rhamnosus* | *Lactobacillus* | *rhamnosus* | CIRM-BIA783 | L_rha_783 | 1 | 1 | 0 | 0 | 1 | 1 | 1 | | 2.2 | 3.6 | C6 |
| *Lacticaseibacillus* | *rhamnosus* | *Lactobacillus* | *rhamnosus* | CIRM-BIA784 | L_rha_784 | 1 | 1 | 0 | 0 | 1 | 1 | 1 | | 1.5 | 2.1 | C6 |
| *Lacticaseibacillus* | *rhamnosus* | *Lactobacillus* | *rhamnosus* | CIRM-BIA1112 | L_rha_1112 | 1 | 1 | 0 | 0 | 1 | 1 | 1 | | 4.1 | 4.2 | C6 |
| *Lacticaseibacillus* | *rhamnosus* | *Lactobacillus* | *rhamnosus* | CIRM-BIA1113 | L_rha_1113 | 1 | 1 | 0 | 0 | 1 | 1 | 1 | | 1 | 1.9 | C6 |
| *Lacticaseibacillus* | *casei* | *Lactobacillus* | *zeae* | CIRM-BIA525 | L_zea_525 | 1 | 1 | 0 | 0 | 1 | 1 | 0 | | 2.1 | 4.6 | C6 |
| *Lactococcus* | *lactis (lactis)* |  |  | S86 | L_lac_477 | 1 | 1 | 0 | 0 | 1 | 1 | 1 | | 1.8 | 1.5 | C6 |
| *Lactococcus* | *lactis (lactis)* |  |  | EIP19F | L_lac_559 | 1 | 1 | 0 | 0 | 1 | 1 | 1 | | 2.2 | 1.6 | C6 |
| *Lactococcus* | *lactis (lactis)* |  |  | CIRM-BIA239 | L_lac_ 239 | 1 | 1 | 0 | 0 | 1 | 1 | 1 | | 2.6 | 2.8 | C6 |
| *Lactococcus* | *lactis (lactis)* |  |  | CIRM-BIA247 | L_lac_247 | 1 | 1 | 0 | 0 | 1 | 1 | 1 | | 2.0 | 3.6 | C6 |
| *Lactococcus* | *lactis (lactis)* |  |  | CIRM-BIA241 | L_lac_ 241 | 1 | 1 | 0 | 0 | 1 | 1 | 1 | | 2.6 | 2.9 | C6 |
| *Lactococcus* | *lactis (lactis)* |  |  | CIRM-BIA242 | L_lac_ 242 | 1 | 1 | 0 | 0 | 1 | 1 | 1 | | 2.6 | 3.1 | C6 |
| *Lactococcus* | *lactis (lactis)* |  |  | CIRM-BIA244 | L_lac_244 | 1 | 1 | 0 | 0 | 1 | 1 | 1 | | 2.7 | 3.4 | C6 |
| *Lactococcus* | *lactis (lactis)* |  |  | CIRM-BIA235 | L_lac_235 | 1 | 1 | 0 | 0 | 1 | 1 | 1 | | 2.4 | 2.8 | C6 |
| *Lacticaseibacillus* | *paracasei* | *Lactobacillus* | *paracasei (paracasei)* | CIRM-BIA1517 | L_par_1517 | 0 | 1 | 0 | 0 | 1 | 1 | 1 | | 0.03 | 0.86 | C7 |
| *Lactococcus* | *lactis (lactis)* |  |  | S188 | L_lac_484 | 0 | 1 | 0 | 0 | 1 | 1 | 1 | | 0.1 | -2.3 | C7 |
| *Lactococcus* | *lactis (lactis)* |  |  | NCDO1867 | L_lac_492 | 0 | 1 | 0 | 0 | 1 | 1 | 1 | | -0.4 | -1.1 | C7 |
| *Lactococcus* | *lactis (lactis)* |  |  | S170 | L_lac_480 | 0 | 1 | 0 | 0 | 1 | 1 | 1 | | -0.4 | -2.1 | C7 |
| *Lactococcus* | *lactis (lactis)* |  |  | UCMA5716 | L_lac_433 | 0 | 1 | 0 | 0 | 1 | 1 | 1 | | -0.2 | -1.5 | C7 |
| *Paralactobacillus* | *sakei* | *Lactobacillus* | *sakei (sakei)* | CIRM-BIA467 | L_sak_467 | 0 | 1 | 0 | 0 | 1 | 0 | 1 | | -0.1 | -0.2 | C7 |
| *Paralactobacillus* | *sakei* | *Lactobacillus* | *sakei (sakei)* | CIRM-BIA1912 | L_sak_1912 | 0 | 1 | 0 | 0 | 1 | 1 | 0 | | 0.1 | -0.0 | C7 |
| *Lacticaseibacillus* | *paracasei* | *Lactobacillus* | *paracasei* | CIRM-BIA279 | L_par_279 | 1 | 1 | 0 | 0 | 1 | 1 | 1 | | 0.5 | 1.6 | C8 |
| *Lacticaseibacillus* | *paracasei* | *Lactobacillus* | *paracasei* | CIRM-BIA281 | L_par_281 | 1 | 1 | 0 | 0 | 1 | 1 | 1 | | 0.5 | 1.2 | C8 |
| *Lacticaseibacillus* | *paracasei* | *Lactobacillus* | *paracasei* | CIRM-BIA283 | L_par_283 | 1 | 1 | 0 | 0 | 1 | 1 | 1 | | 0.1 | 1.3 | C8 |
| *Lacticaseibacillus* | *paracasei* | *Lactobacillus* | *paracasei* | CIRM-BIA285 | L_par_285 | 1 | 1 | 0 | 0 | 1 | 1 | 1 | | 0.1 | 1.1 | C8 |
| *Lacticaseibacillus* | *paracasei* | *Lactobacillus* | *paracasei* | CIRM-BIA286 | L_par_286 | 1 | 1 | 0 | 0 | 1 | 1 | 1 | | 0.2 | 1.6 | C8 |
| *Lacticaseibacillus* | *paracasei* | *Lactobacillus* | *paracasei* | CIRM-BIA287 | L_par_287 | 1 | 1 | 0 | 0 | 1 | 1 | 1 | | 0.1 | 1.5 | C8 |
| *Lacticaseibacillus* | *paracasei* | *Lactobacillus* | *paracasei* | CIRM-BIA288 | L_par_288 | 1 | 1 | 0 | 0 | 1 | 1 | 1 | | -0.1 | 0.6 | C8 |
| *Lacticaseibacillus* | *paracasei* | *Lactobacillus* | *paracasei* | CIRM-BIA290 | L_par_290 | 1 | 1 | 0 | 0 | 1 | 1 | 1 | | 0.3 | 1.7 | C8 |
| *Lacticaseibacillus* | *paracasei* | *Lactobacillus* | *paracasei* | CIRM-BIA291 | L_par_291 | 1 | 1 | 0 | 0 | 1 | 1 | 1 | | -0.1 | 1.1 | C8 |
| *Lactiplantibacillus* | *paraplantarum* | *Lactobacillus* | *paraplantarum* | CIRM-BIA778 | L_par_778 | 1 | 1 | 0 | 0 | 1 | 1 | 1 | | -0.2 | -0.4 | C8 |
| *Lactiplantibacillus* | *paraplantarum* | *Lactobacillus* | *paraplantarum* | CIRM-BIA779 | L_par_779 | 1 | 1 | 0 | 0 | 1 | 1 | 1 | | -0.3 | -0.4 | C8 |
| *Lactiplantibacillus* | *pentosus* | *Lactobacillus* | *pentosus* | CIRM-BIA660 | L_pen_660 | 1 | 1 | 0 | 0 | 1 | 1 | 1 | | 0.0 | 0.1 | C8 |
| *Lactiplantibacillus* | *plantarum* | *Lactobacillus* | *plantarum* | CIRM-BIA1111 | L_pla_1111 | 1 | 1 | 0 | 0 | 1 | 1 | 1 | | -0.1 | 0.5 | C8 |
| *Lactobacillus* | *johnsonii* | *Lactobacillus* | *johnsonii* | CIRM-BIA873 | L_joh_873 | 1 | 1 | 0 | 0 | 1 | 1 | 1 | | 0.6 | 0.8 | C8 |
| *Lactococcus* | *lactis (lactis)* |  |  | NCDO2146 | L_lac_497 | 1 | 1 | 0 | 0 | 1 | 1 | 1 | | 0.2 | -0.5 | C8 |
| *Lactococcus* | *lactis (lactis)* |  |  | EIP20A | L_lac_632 | 1 | 1 | 0 | 0 | 1 | 1 | 1 | | 0.0 | -0.8 | C8 |
| *Lactococcus* | *lactis (lactis)* |  |  | EIP13G | L_lac_537 | 1 | 1 | 0 | 0 | 1 | 1 | 1 | | 0.1 | -1.0 | C8 |
| *Lactococcus* | *lactis (lactis)* |  |  | EIP03B | L_lac_621 | 1 | 1 | 0 | 0 | 1 | 1 | 1 | | -0.2 | -1.2 | C8 |
| *Paralactobacillus* | *sakei* | *Lactobacillus* | *sakei* | CIRM-BIA1559 | L_sak_1559 | 1 | 1 | 0 | 0 | 1 | 1 | 0 | | -0.1 | -0.0 | C8 |
| *Paralactobacillus* | *sakei* | *Lactobacillus* | *sakei (sakei)* | CIRM-BIA892 | L_sak_892 | 1 | 1 | 0 | 0 | 1 | 1 | 0 | | 0.1 | 1.5 | C8 |

*^a^* Collections: CIRM-BIA : International Centre for Microbial Resources dedicated to bacteria of food interest, INRAE Rennes, France, https://www6.rennes.inrae.fr/stlo_eng/, NCDO :National Collection of Dairy Organisms; now NC of Food Bacteria, Berkshire UK, UCMA: Université de Caen – Microbiologie alimentaire, Caen, France, and LBAE: Laboratoire de Biotechnologies Agroalimentaire et Environnementale, Auch, France

*^b^* Carbohydrate fermentation was evaluated as a binary trait, using API 50CH gallery (Biomerieux): a value of 1 indicates that the carbohydrate is degraded. LAC = lactose; STA = Stachyose; RAF = Raffinose; SUC = Sucrose; FRU = Fructose; GAL = Galactose; MAL = Maltose. Nd: not determined

*^c^* Proteolytic indices determined by OPA method and expressed as the difference between the value of the sample after 48 h incubation minus the value of the control incubated under the same conditions (unit is mM equivalent methionine used as a standard). The mean value of the medium incubated without bacteria, *i.e.* the control, was 3.1 ± 0.4 mM eq. Met with caseins and 3.4 ± 0.4 mM eq. Met with lupin proteins.

*^d^* Cluster: see figure 1.

**Table S4**: Volatile compounds identified in the fermented milk-lupin mixes (MLM) after 24 h fermentation and their corresponding origin and flavor type

| Compound (trivial name)*^a^* | m/z *^b^* | Identification *^c^* | LRI *^d^* | Origin *^e^* | Flavor type *^f^* | P-anova *^g^* | Max ratio  between  cultures/unfermented MLM^h^ |
| --- | --- | --- | --- | --- | --- | --- | --- |
| 2 Methylpropanal | 41 | S, LRI, DB | 812 | AA | Aldehydic | 1.3 10^-05^ | 13.0 |
| 2-Propanone (acetone) | 58 | LRI, DB | 811 | C | Solvent | 0.74 | 1.4 |
| Ethyl acetate | 61 | S, LRI, DB | 876 | C | Ethereal | 0.42 | 9.7 |
| 2-Butanone | 43 | S, LRI, DB | 890 | unk | Chemical | 0.48 | 4.0 |
| 3-Methylbutanal | 58 | S, LRI, DB | 902 | AA | Fruity | 9.1 10^-06^ | 244.4 |
| 2-Pentanone | 71 | LRI, DB | 961 | unk | Fruity | 9.2 10^-05^ | 6.8 |
| 2,3-Butanedione (diacetyl) | 86 | S, LRI, DB | 970 | C | Buttery | 7.7 10^-16^ | 298.2 |
| Ethyl butanoate | 71 | S, LRI, DB | 1032 | C | Fruity | 2.3 10^-02^ | 1.7 |
| 2,3-Pentanedione | 100 | S, LRI, DB | 1067 | AA | Toasted | 4.2 10^-06^ | 67.4 |
| Dimethyl disulfide | 94 | S, LRI, DB | 1065 | AA | Sulfurous | 1.1 10^-02^ | 120.2 |
| hexanal | 72 | LRI, DB | 1076 | unk | Green | 4.7 10^-13^ | 1.0 |
| 112-Heptanone | 58 | S, LRI, DB | 1178 | FFA | Cheesy | 2.3 10^-08^ | 10.4 |
| 2-Pentylfuran | 82 | LRI, DB | 1203 | V | Green | 0.81 | 1.6 |
| 2-Methyl- and 3-methylbutanol | 55 | S, LRI, DB | 1216 | AA | Fusel | 1.2 10^-10^ | 721.3 |
| 3,2-Hydroxybutanone (acetoin) | 45 | S, LRI, DB | 1274 | C | Creamy | 3.4 10^-11^ | 236.6 |
| 1-Hydroxy-2-propanone | 74 | LRI, DB | 1293 | unk | Sweet | 1.0 10^-02^ | 11.6 |
| 2-Nonanone | 58 | S, LRI, DB | 1378 | FFA | Cheesy | 1.210^-18^ | 102.0 |
| 1-Hexanol | 56 | LRI, DB | 1355 | FFA | Green | 1.110^-02^ | 5.8 |
| Acetic acid | 60 | S, LRI, DB | 1451 | C | Sour | 1.110^-05^ | 13.5 |
| Benzaldehyde | 106 | S, LRI, DB | 1511 | AA | Fruity | 3.5 10^-13^ | 1 |
| 2-Undecanone | 58 | LRI, DB | 1588 | FFA | Waxy | 1.6 10^-08^ | 27.8 |
| Butanoic acid | 60 | S, LRI, DB | 1624 | L | Sour | 7.3 10^-08^ | 25.5 |
| 2-Phenylacetaldehyde | 91 | S, LRI, DB | 1634 | AA | Honey | 5.6 10^-19^ | 451.1 |
| 2-Methylbutanoic | 74 | S, LRI, DB | 1674 | AA | Fruity | 2.6 10^-02^ | 76.5 |
| Hexanoic acid | 60 | S, DB | Nd | L | Cheesy | 1.4 10^-08^ | 19.2 |
| Phenylethanol | 91 | DB | Nd | AA | Floral | 1.5 10^-12^ | 113.5 |
| Octanoic acid | 60 | S, DB | Nd | L | Soapy | 1.9 10^-04^ | 10.4 |
|  |  |  |  |  |  |  |  |

*^a^* Compound name according to IUPAC (International Union of Pure and Applied Chemistry) nomenclature followed by trivial name.

*^b^* m/z, mass fragment used for quantification.

*^c^* Compounds identified based on: S, Retention time and mass spectrum from standard; LRI, linear retention index; DB, mass spectral data Library NIST;

*^d^* value of the linear retention index (LRI)

*^e^* Probable pathway of origin: L, lipolysis, C, carbohydrate metabolism, AA, amino acid metabolism, FFA, free fatty acid metabolism; unk, unknown.

*^f^* Flavor type according to the <http://thegoodscentscompany.com/>

*^g^* P-value of ANOVA according to the modality of each type of cultures (mono- and co-cultures)

*^h^* Ratio of maximal concentrations in cultures (mono- and co-cultures) and in control.


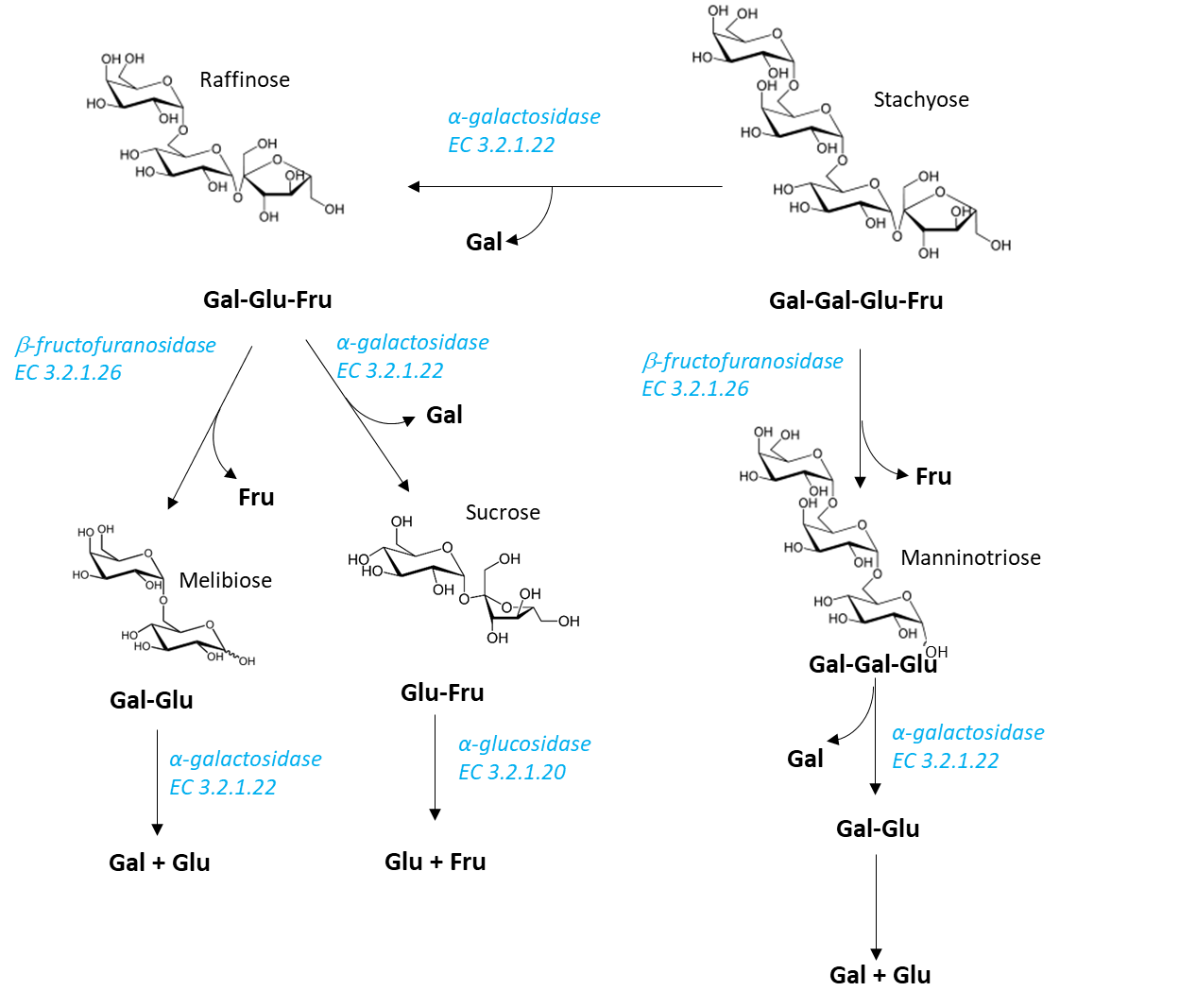


**Figure S1:** Pathways and enzymes implied in the hydrolysis of stachyose and raffinose (extracted from the galactose metabolism of *Lactococcus lactis* in the KEGG database, <https://www.genome.jp/kegg/pathway.html>)

# References

1. Furet J-P, Quénée P, Tailliez P. Molecular quantification of lactic acid bacteria in fermented milk products using real-time quantitative PCR. *Int J Food Microbiol* 2004; **97**: 197–207.

2. Falentin H, Postollec F, Parayre S, Henaff N, Le Bivic P, Richoux R, et al. Specific metabolic activity of ripening bacteria quantified by real-time reverse transcription PCR throughout Emmental cheese manufacture. *Int J Food Microbiol* 2010; **144**: 10–19.

3. Achilleos C, Berthier F. Quantitative PCR for the specific quantification of *Lactococcus lactis* and *Lactobacillus paracasei* and its interest for *Lactococcus lactis* in cheese samples. *Food Microbiol* 2013; **36**: 286–295.

4. Torriani S, Felis GE, Dellaglio F. Differentiation of *Lactobacillus plantarum*, *L. pentosus*, and *L. paraplantarum* by recA gene sequence analysis and multiplex PCR assay with recA gene-derived primers. *Appl Environ Microbiol* 2001; **67**: 3450–3454.

5. Zheng J, Wittouck S, Salvetti E, Franz CMAP, Harris HMB, Mattarelli P, et al. A taxonomic note on the genus Lactobacillus: Description of 23 novel genera, emended description of the genus *Lactobacillus Beijerinck* 1901, and union of Lactobacillaceae and Leuconostocaceae. *Int J Syst Evol Microbiol* 2020; **70**.
